# Integrative transcriptomic and single-cell analysis identifies anoikis-related molecular signatures in vascular calcification

**DOI:** 10.1080/0886022X.2026.2704989

**Published:** 2026-08-24

**Authors:** Dandan Chen, Tianzeng Dai, Wenfeng Yu, Xi’na Xie, Rui Li, Yan Wang

**Affiliations:** ^a^ Department of Nephrology, Mengchao Hepatobiliary Hospital of Fujian Medical University, Fuzhou, P.R. China; ^b^ Department of Urology, Mengchao Hepatobiliary Hospital of Fujian Medical University, Fuzhou, P.R. China; ^c^ Blood Purification Center, Mengchao Hepatobiliary Hospital of Fujian Medical University, Fuzhou, P.R. China

**Keywords:** Chronic kidney disease, vascular calcification, anoikis, vascular smooth muscle cells, machine learning, single-cell RNA sequencing

## Abstract

**Background:**

Vascular calcification (VC) is a life-threatening complication of chronic kidney disease (CKD) driven by vascular smooth muscle cell (VSMC) osteogenic transdifferentiation. Anoikis, a form of adhesion-dependent apoptosis, is involved in cardiovascular remodeling, yet its regulatory role in CKD-associated VC remains unexplored.

**Methods:**

We performed an integrative transcriptomic and single‑cell analysis using a human *in vitro* VSMC calcification model and a rat *in vivo* CKD‑VC model. Differentially expressed genes were identified under a unified threshold (FDR < 0.05, |log_2_FC| > 1). Strict one‑to‑one ortholog mapping was applied. Differentially expressed anoikis‑related genes were screened, followed by functional enrichment, three machine‑learning algorithms, and external validation.

**Results:**

We identified 48 differentially expressed anoikis‑related genes, with the signal derived mainly from human VSMCs rather than cross‑species conservation. The cell adhesion molecule pathway was significantly dysregulated. Seven hub genes were identified: BDNF, CRYAB, CYP1B1, DAPK1, HAS2, PDGFRB, and PLAU. The seven‑gene model achieved an AUC of 0.811 in the independent validation cohort. Single‑cell analysis revealed cell‑type‑specific expression patterns, with the highest anoikis module scores in osteoblast‑like cells and macrophages.

**Conclusions:**

This study identifies novel anoikis‑related molecular signatures associated with VC. These genes are involved in cell adhesion, VSMC homeostasis, chaperone‑mediated cytoprotection, and matrix remodeling. Our exploratory findings provide new insights into VC pathogenesis and require further functional and clinical validation in CKD‑specific cohorts.

## 1. Introduction

Chronic kidney disease (CKD) affects approximately 10–15% of the global population and remains the leading non-communicable disease driver of cardiovascular mortality [1,2]. Among the diverse cardiovascular complications of CKD, vascular calcification (VC) stands out as one of the most prevalent and clinically consequential, afflicting over 80% of dialysis patients and independently predicting all-cause death [3,4]; in the general population the coronary artery calcium score is likewise an established independent predictor of cardiovascular events [5]. Traditionally regarded as a passive degenerative process, VC is now recognized as an actively regulated, cell-mediated phenomenon involving vascular smooth muscle cell (VSMC) dysfunction, extracellular matrix (ECM) remodeling, and the deposition of hydroxyapatite crystals within the arterial wall [6]. In CKD, disturbed mineral metabolism—particularly hyperphosphatemia—creates a procalcific milieu that drives VSMCs to shed their contractile phenotype and acquire osteoblast-like characteristics, marked by the upregulation of osteogenic transcription factors Runx2 and alkaline phosphatase [7,8]. Classical hypothesis-driven studies in uremic rat models established that 1,25(OH)_2_D_3_ induces systemic cardiovascular disease and aortic medial calcification accompanied by upregulation of osteoblast markers, calcium-transporting proteins, and osterix, framing CKD-VC as an active, cell-mediated osteogenic process [9,10]. Despite advances in phosphate binders and calcimimetics, effective therapies that directly target the molecular mechanisms of VC remain elusive [11].

The phenotypic transition of VSMCs is a pivotal step in CKD-associated VC. Under pathological stimuli such as high phosphate, uremic toxins, and oxidative stress, VSMCs downregulate contractile markers (SM α-actin, SM22α, MYH11) and upregulate osteochondrogenic genes, thereby acquiring the capacity to release calcifying extracellular vesicles and remodel the surrounding matrix [12–14]. Multiple signaling cascades—including PI3K-Akt, MAPK, Wnt/β-catenin, and TGF-β—have been implicated in this phenotypic switch [15,16]. Concurrently, several forms of regulated cell death—apoptosis, autophagy, ferroptosis, and pyroptosis—participate in VC progression; notably, apoptotic bodies from dying VSMCs serve as nucleation sites for mineral deposition [17–19]. Yet the full repertoire of cell-death modalities governing VSMC fate in the calcifying microenvironment remains incompletely mapped.

Anoikis is a specialized form of intrinsic apoptosis triggered by the loss of integrin-dependent cell–ECM adhesion, first described by Frisch and Francis in 1994 [20]. Upon cell detachment, pro-apoptotic Bcl-2 family members (Bax, Bak, Bim) are activated while anti-apoptotic proteins (Bcl-2, Bcl-xL) are suppressed, leading to mitochondrial outer-membrane permeabilization, cytochrome c release, and caspase cascade activation [21,22]. Although anoikis resistance has been extensively studied in cancer metastasis [23], emerging evidence implicates anoikis in pathological cardiovascular remodeling, including smooth muscle cell disappearance in aneurysms, endothelial denudation in atherosclerosis, and cardiomyocyte detachment in heart failure [24,25]. Critically, the plasminogen activation system provides a direct mechanistic link between anoikis and vascular pathology: pericellular plasmin generated by urokinase-type plasminogen activator (uPA/PLAU) on the VSMC surface degrades fibronectin, inducing cell retraction, detachment, and anoikis [26]. Moreover, anoikis resistance—mediated through aberrant activation of FAK, PI3K/Akt, and Ras/ERK pathways—may enable phenotypically altered VSMCs to survive within the remodeled calcifying matrix [23,27]. Despite this rationale, the role of anoikis-related genes (ARGs) in CKD-associated VC has not been systematically investigated.

Single-omics approaches have already substantially advanced CKD research; for example, metabolomics has clarified uremic-toxin biology, altered amino-acid and lipid metabolism, oxidative stress, and biomarker discovery in CKD [28]. However, a single transcriptomic layer cannot determine whether expression changes translate into protein activity, ECM remodeling, or functional calcification, and cannot localize signals to specific cell types. Integrating bulk and single-cell transcriptomic data can connect upstream regulation to cell-type-resolved programmes, prioritize candidates by cross-dataset consistency, and place genes within a broader disease architecture. We therefore describe the present work as an integrative transcriptomic and single-cell analysis rather than a multi-omics study, since no proteomic, metabolomic, epigenomic, or spatial layers were integrated.

Recent bioinformatics studies have explored anoikis-related signatures using machine-learning frameworks, primarily in cancer [29,30] and, more recently, in diabetic nephropathy [31], and diagnostic models for VC in diabetic kidney disease have been constructed using similar approaches [32]. To our knowledge, however, no prior study has focused specifically on the anoikis–CKD-VC intersection. Whereas classical hypothesis-driven uremic models [9,10] necessarily test pre-selected osteogenic pathways, an integrative data-driven approach can surface non-canonical programmes—cell–matrix adhesion, proteolytic remodeling, and anoikis susceptibility—that targeted studies are less likely to uncover. In the present study we identify and characterize anoikis-related molecular signatures in phosphate-driven vascular calcification, using GSE146638 (rat CKD-VC RNA-seq) and GSE37558 (human VSMC high-phosphorus microarray) for discovery, machine learning for hub-gene prioritization, external validation in GSE43292, and single-cell transcriptomics (GSE159677) for cell-type-resolved contextualization.

## 2. Materials and methods

### 2.1. Data acquisition and preprocessing

Gene expression datasets were retrieved from the Gene Expression Omnibus (GEO). A human in-vitro VSMC discovery dataset (GSE37558) and a rat in-vivo CKD-VC dataset (GSE146638), the latter used as an independent in-vivo cross-check rather than a parallel training cohort, were analyzed (Table 1). GSE146638 is an RNA-seq dataset profiling aortic tissues from a uremic rat (Rattus norvegicus, genome rn5) model of CKD-associated vascular calcification, including uremic and vitamin D3-induced calcification groups versus normal controls (Control = 5, Disease = 9). The GEO supplementary file provides only RPKM values (Cufflinks/Cuffdiff output); raw counts were not deposited. To enable a count-based analysis, raw single-end reads were downloaded from SRA (study SRP252073; 30 runs corresponding to the 14 samples), adapter- and quality-trimmed with fastp, quantified against the Ensembl release-110 rat transcriptome with salmon [33], and summarized to gene-level counts with tximport, yielding 29,910 genes. Rat gene symbols were mapped to human orthologs using biomaRt (Ensembl release 110) [34], retaining one-to-one orthologs with orthology confidence = 1; concordance with NCBI HomoloGene was 99.0%. GSE37558 is a microarray dataset (Affymetrix HG-U133 Plus 2.0, GPL570) profiling human VSMCs cultured under high-phosphorus calcification-inducing conditions (calcifying vascular cells, CVC) versus undifferentiated day-0 VSMCs (VSMC d0 = 4, CVC = 12; 25,159 genes after probe deduplication). An overview of the full analytical pipeline is provided in Supplementary Figure S0, and the complete list of CKD-VC datasets considered, with inclusion and exclusion reasons, is given in Supplementary Table S1.

**Table 1.** Characteristics of the GEO datasets used in this study.

Dataset	Description	Species	Platform	Sample size	Role
GSE146638	CKD aortic VC (uremic + VitD3)	Rat	RNA-seq (re-quantified from SRA)	Ctrl = 5, Dis = 9	In-vivo cross-check (non-confirmatory)
GSE37558	High-Pi human VSMC calcification	Human	Microarray (GPL570)	VSMC = 4, CVC = 12	Training (DEG+ML+immune)
GSE43292	Human carotid atherosclerosis (paired)	Human	Microarray (HuGene 1.0 ST)	Intact = 32, Plaque = 32	External validation
GSE159677	Human carotid plaque scRNA-seq	Human	10X Genomics	47,861 cells (post-QC)	Single-cell contextualization

Species of GSE146638 corrected to rat; GSE146638 re-quantified from SRA; single-cell cell count updated to 47,861.

For external validation, GSE43292 (Affymetrix Human Gene 1.0 ST) was used, comprising paired carotid atherosclerotic plaque and intact tissue from 32 patients (*n* = 64; intact = Control, plaque = Disease). Gene symbols were parsed from the probe annotation (second ‘//’-delimited field) and collapsed per symbol by retaining the highest-mean probe. For single-cell contextualization, GSE159677 (10X Genomics scRNA-seq of human carotid atherosclerotic plaques) was used. All datasets were public; no ethical approval was required.

### 2.2. Differential expression analysis

For GSE146638, gene-level counts were filtered with filterByExpr (12,819 genes retained) [35], TMM-normalized, and analyzed with limma-voom and empirical Bayes moderation [36]. For GSE37558, log2 expression was analyzed with limma. A single unified threshold—adjusted *p* < 0.05 (Benjamini–Hochberg) and |log2FC| > 1—was applied to both datasets, replacing the previous dataset-specific thresholds. This yielded 153 DEGs in GSE146638 and 690 DEGs in GSE37558. As a sanity check, DESeq2 [37] on the same counts recovered 88% of the voom DEGs for GSE146638. Volcano plots were generated using ggplot2.

### 2.3. Construction of the anoikis-related gene set

A comprehensive ARG set was compiled from two sources. First, four GO:BP anoikis terms (GOBP_ANOIKIS, GOBP_NEGATIVE/POSITIVE/REGULATION_OF_ANOIKIS) from MSigDB *via* msigdbr (36 unique genes; the canonical core). Second, GeneCards keyword ‘anoikis’ genes with relevance score > 1.0 (∼525 genes). After deduplication, the final ARG set comprised 529 genes (full list with per-gene source annotation and relevance score is provided as Supplementary Table S2). Because the panel genes derive from the broader GeneCards list rather than the 36 GO-core genes, sensitivity analyses restricting to the GO core (and to a stricter GeneCards score > 7.0) were performed (Supplementary §S3). The corresponding ARG-set sensitivity-analysis results are shown in Supplementary Figure S3.

### 2.4. Identification of DEARGs

DEARGs were identified by intersecting the human-mapped DEGs from each dataset with the 529-gene ARG set; the union was used downstream and genes shared by both datasets were recorded as a common (cross-species) set. Venn diagrams used the VennDiagram package.

### 2.5. Functional enrichment analysis

GO (BP/MF/CC) and KEGG over-representation analysis on the union DEARGs used clusterProfiler [38] with org.Hs.eg.db (Benjamini–Hochberg adjusted *p* < 0.05).

### 2.6. Gene set enrichment analysis (GSEA)

GSEA [39] was performed on the genome-wide GSE37558 profile, ranking genes by the moderated t-statistic and using gseGO and gseKEGG (adjusted *p* < 0.05).

### 2.7. Gene set variation analysis (GSVA)

selected for established relevance to CKD, VC, apoptosis and adhesion: KEGG_APOPTOSIS, KEGG_CALCIUM_SIGNALING_PATHWAY, KEGG_CELL_ADHESION_MOLECULES_CAMS, KEGG_CYTOKINE_CYTOKINE_RECEPTOR_INTERACTION, KEGG_ECM_RECEPTOR_INTERACTION, KEGG_FOCAL_ADHESION, KEGG_JAK_STAT_SIGNALING_PATHWAY, KEGG_MAPK_SIGNALING_PATHWAY, KEGG_NOTCH_SIGNALING_PATHWAY, KEGG_P53_SIGNALING_PATHWAY, KEGG_MTOR_SIGNALING_PATHWAY (used in place of KEGG_PI3K_AKT_SIGNALING_PATHWAY, which is absent from the MSigDB KEGG-legacy collection), KEGG_REGULATION_OF_ACTIN_CYTOSKELETON, KEGG_TGF_BETA_SIGNALING_PATHWAY, KEGG_VEGF_SIGNALING_PATHWAY, and KEGG_WNT_SIGNALING_PATHWAY (also listed in Supplementary Table S5). Differential pathway activity was assessed with limma.

### 2.8. Immune-related gene-programme analysis (ssGSEA)

Immune-related gene-programme activity in GSE37558 was estimated using ssGSEA (GSVA package) with marker sets for 18 immune cell types from Charoentong et al. [41]. Because GSE37558 is an in-vitro VSMC monoculture devoid of immune cells, these scores reflect immune-related gene-expression programmes within VSMCs, not immune cell infiltration; the analysis is presented in Supplementary §S5 and interpreted strictly as gene-programme trends. Differences used Welch’s t-test with Benjamini–Hochberg correction.

### 2.9. Machine-learning hub-gene selection

Three algorithms were applied to the union DEARGs using GSE37558 (*n* = 16). To avoid the asymmetry of the original analysis (in which LASSO retained 2, SVM-RFE 5, and random forest 67 genes), a pre-specified symmetric rule was used: each algorithm contributed its top 10 candidates—LASSO by |coefficient| at λ1se [42], random forest by Mean Decrease Accuracy [43], and SVM-RFE by RFE rank—and hub genes were defined as those selected by ≥ 2 of 3 algorithms. The complete SVM-RFE ranking is provided as Supplementary Table S4. Seed = 42.

### 2.10. Diagnostic model construction and validation

A logistic regression model used the hub genes as predictors (GSE37558 training; glm, binomial). Expression was z-score standardized within each dataset before coefficient transfer. Discrimination was quantified by AUC with DeLong 95% confidence intervals (pROC) [44]; internal optimism was assessed by repeated 5-fold cross-validation and 1,000-replicate bootstrap optimism correction, and calibration by the Hosmer–Lemeshow test. The model was externally validated in GSE43292, and single-gene ROC curves were computed.

### 2.11. Hub gene–immune correlation

Spearman correlations between hub-gene expression and ssGSEA immune-programme scores across the 16 GSE37558 samples were visualized with corrplot (non-significant masked).

### 2.12. Protein–protein interaction (PPI) network

The 48 union DEARGs were submitted to STRING v12.0 (Homo sapiens; confidence ≥ 0.400). Genes lacking any medium-confidence interaction were recorded as network attrition.

### 2.13. Single-cell RNA sequencing analysis

GSE159677 was processed with Seurat v4 [45] (QC: 200–5,000 genes, >500 UMI, <15% mitochondrial reads; LogNormalize; 2,000 variable genes; PCA; SNN; Louvain resolution 0.5; UMAP). Clusters were annotated by canonical lineage markers and cross-referenced with published plaque atlases [46]. After QC, 47,861 cells were annotated into 18 cell types. Anoikis activity was scored with AddModuleScore using ARGs detected in the dataset.

### 2.14. Statistical analysis

Analyses used R 4.5. Multiple testing used Benjamini–Hochberg FDR; correlations used Spearman’s method; tests were two-sided with *p* < 0.05 considered significant. All analytic code and intermediate objects are deposited at a public repository (GitHub/Zenodo; see Data and Code Availability). Random seeds were 42.

## 3. Results

### 3.1. Identification of DEGs and DEARGs

**Figure 1.**
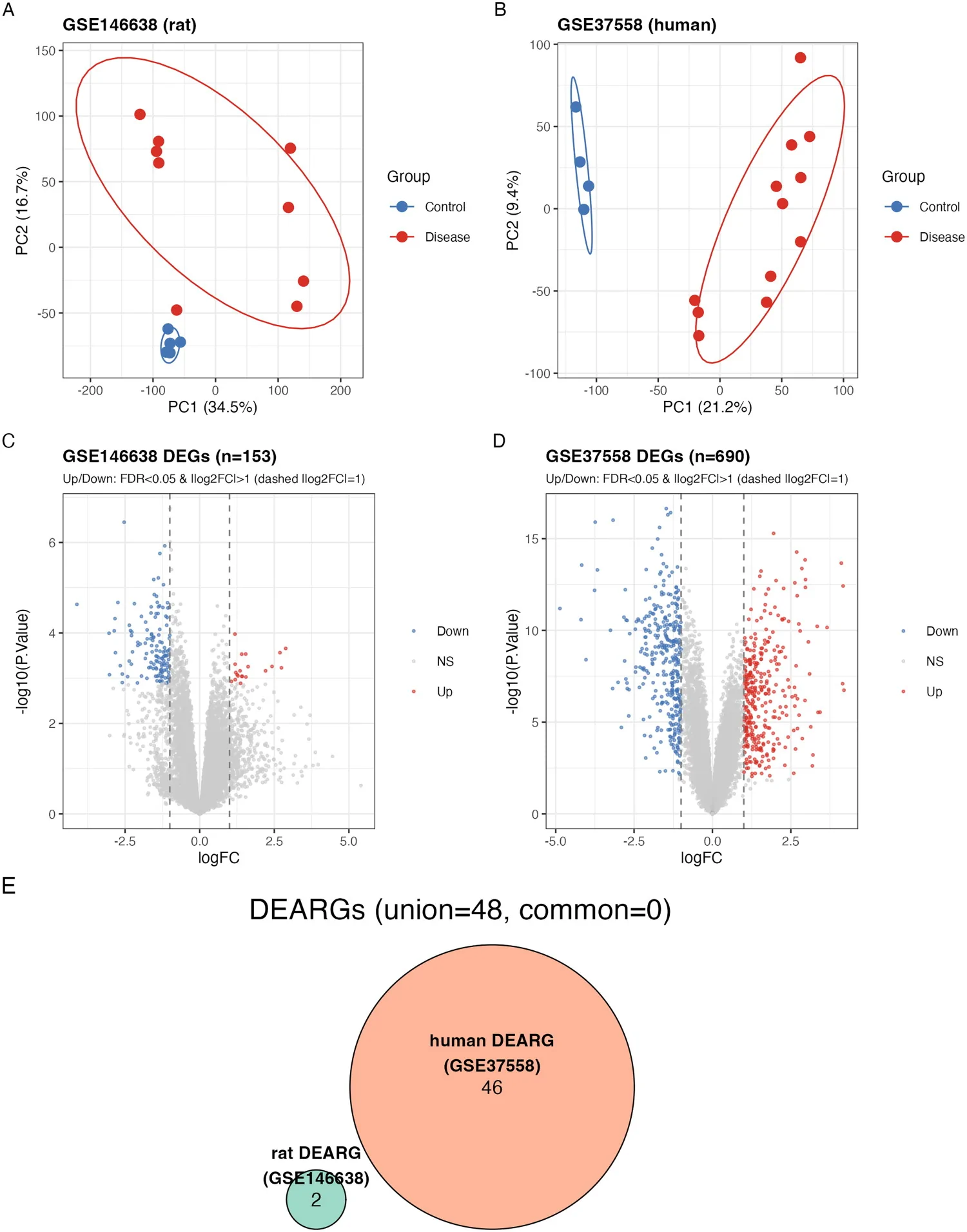
Identification of DEGs and DEARGs. (A–B) PCA of GSE146638 (rat CKD-VC) and GSE37558 (human VSMC). (C) Volcano plot of GSE146638 (voom; FDR < 0.05, |log_2_FC| > 1; 153 DEGs). (D) Volcano plot of GSE37558 (690 DEGs). In C–D, the y-axis is − log10(P.Value) for visualization, whereas Up/Down coloring denotes significance defined on FDR (FDR < 0.05 and |log2FC| > 1); vertical dashed lines mark |log2FC| = 1. (E) Venn diagram of human-mapped DEGs intersected with the 529-gene ARG set: 2 DEARGs from GSE146638 and 46 from GSE37558, union = 48, with no common cross-species gene.

Under the unified threshold (FDR < 0.05, |log2FC| > 1), differential expression identified 153 DEGs in GSE146638 (voom; 88% concordant with DESeq2) and 690 DEGs in GSE37558. Principal-component analysis separated Disease from Control samples in both discovery datasets (Figure 1A,B), and volcano plots illustrate the corresponding expression landscapes (Figure 1C,D).

Mapping the 153 rat DEGs to human orthologs (115 high-confidence one-to-one, 75%) and intersecting with the 529-gene ARG set yielded 2 DEARGs from GSE146638 and 46 from GSE37558, for a union of 48 unique DEARGs. Notably, the cross-species common set was empty (0 genes). Formal enrichment testing showed that the rat in-vivo dataset was not enriched for ARGs (2 observed vs 3.2 expected; hypergeometric *p* = 0.84), whereas the human in-vitro dataset was strongly enriched (46 vs 18.3 expected; *p* = 9.5 × 10^−9^). The anoikis-related signal therefore derives essentially from the human phosphate-driven VSMC model; the apparent cross-species conservation reported previously was not reproduced under count-based voom analysis and rigorous orthology mapping (Figure 1E). The complete union, rat-derived, and human-derived DEARG lists are provided in Supplementary Table S3.

### 3.2. Functional enrichment of DEARGs

**Figure 2.**
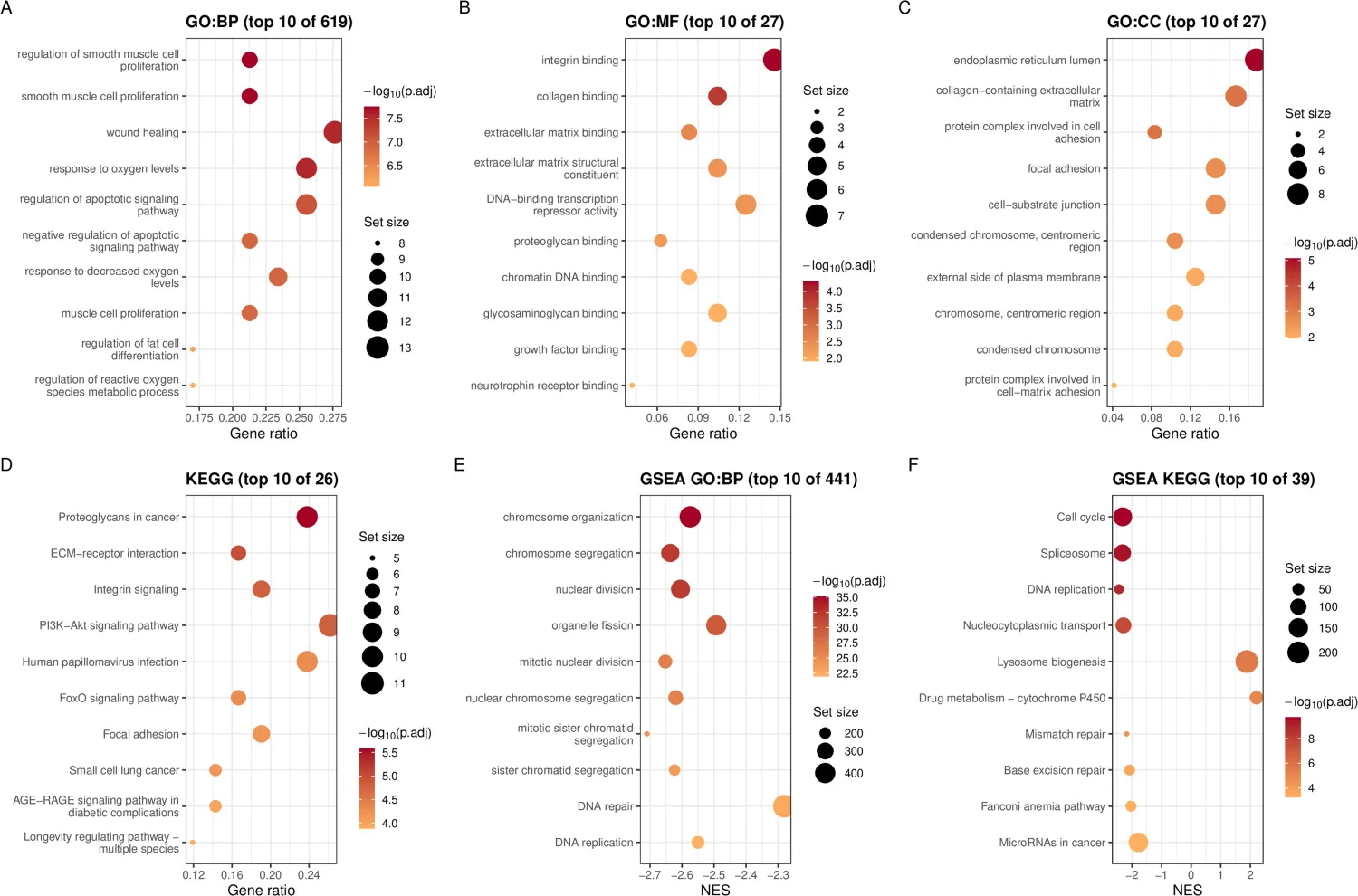
Functional enrichment of the 48 union DEARGs. (A) GO:BP dot plot (top terms: regulation of smooth muscle cell proliferation, wound healing, apoptotic signaling). (B–C) GO MF/CC dot plots. (D) KEGG dot plot. (E–F) GSEA GO:BP and KEGG dot plots from the full GSE37558 transcriptome (441 GO and 39 KEGG terms significant).

GO enrichment of the 48 union DEARGs revealed 619 significant BP terms, the most enriched being regulation of smooth muscle cell proliferation, wound healing, response to oxygen levels, and regulation of apoptotic signaling pathway (Figure 2A). At the molecular-function and cellular-component levels, the DEARGs were enriched for integrin binding, collagen binding and extracellular-matrix binding, and localized to the collagen-containing extracellular matrix, focal adhesions and cell–substrate junctions (Figure 2B,C). KEGG analysis identified 26 enriched pathways, led by proteoglycans in cancer, ECM–receptor interaction, integrin signaling, PI3K–Akt signaling and focal adhesion (Figure 2D). The convergence of apoptotic, adhesion, and survival signaling among DEARGs is consistent with a role for anoikis-related programmes in vascular calcification.

### 3.3. GSEA reveals global pathway perturbation

GSEA of the full GSE37558 transcriptome identified 441 significant GO:BP terms and 39 significant KEGG pathways. Cell-cycle and mitotic processes were prominent among activated terms, indicating proliferative reprogramming during VSMC calcification (Figure 2E,F).

### 3.4. GSVA identifies cell adhesion molecules as the most altered pathway

**Figure 3.**
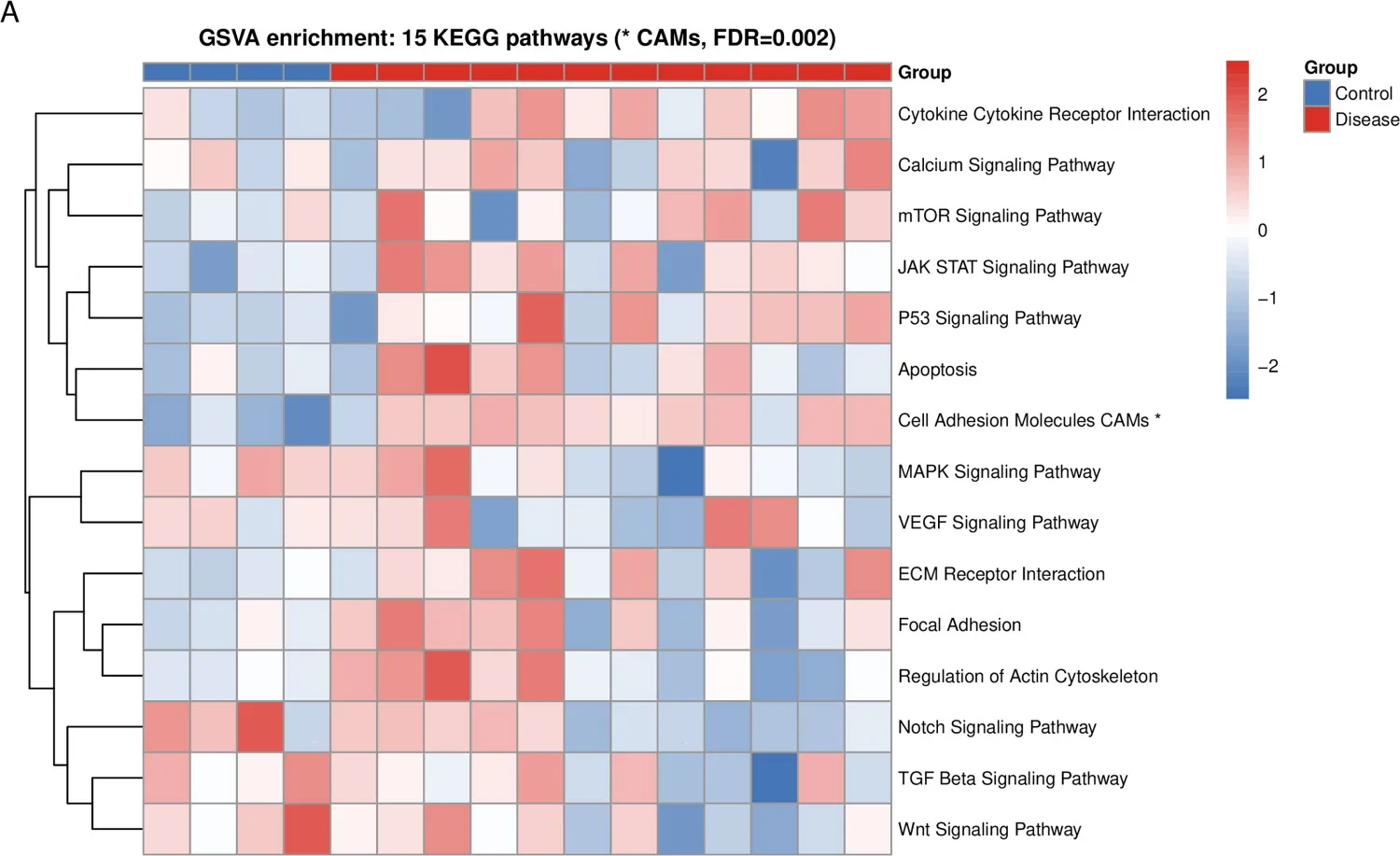
Pathway analysis in GSE37558. (A) GSVA heatmap of the 15 prioritized KEGG pathways (rows in Title case, KEGG_ prefix removed; columns ordered and annotated by group). Cell Adhesion Molecules (CAMs) was the only pathway significant after FDR correction (adjusted *p* = 0.002) and is marked with an asterisk (*); all other pathways showed nominal trends only (adjusted *p* ≥ 0.20). The ssGSEA immune-related gene-programme analysis, for which no signature survived FDR correction, is now presented separately in Supplementary Figure S5.

Among the 15 KEGG pathways, KEGG_CELL_ADHESION_MOLECULES_CAMS was the only pathway significant after FDR correction (logFC = 0.249, adjusted *p* = 0.002), with elevated activity in the Disease group (Figure 3A). Notch and p53 signaling showed nominal (uncorrected) trends only and were non-significant after FDR (adjusted *p* = 0.20). The predominant alteration of cell adhesion molecule activity is directly relevant to anoikis, as integrin-mediated cell–ECM adhesion is the primary determinant of anoikis sensitivity.

### 3.5. Immune-related transcriptomic signature analysis

ssGSEA of immune-related gene programmes in GSE37558 showed only nominal (uncorrected) trends (e.g., Th2 and pDC programmes); no signature survived FDR correction. Because this dataset is an in-vitro monoculture, these results reflect immune-related gene-expression reprogramming within VSMCs rather than immune cell infiltration, and are presented in Supplementary §S5 (Supplementary Figure S5).

### 3.6. Machine learning identifies seven hub genes

**Figure 4.**
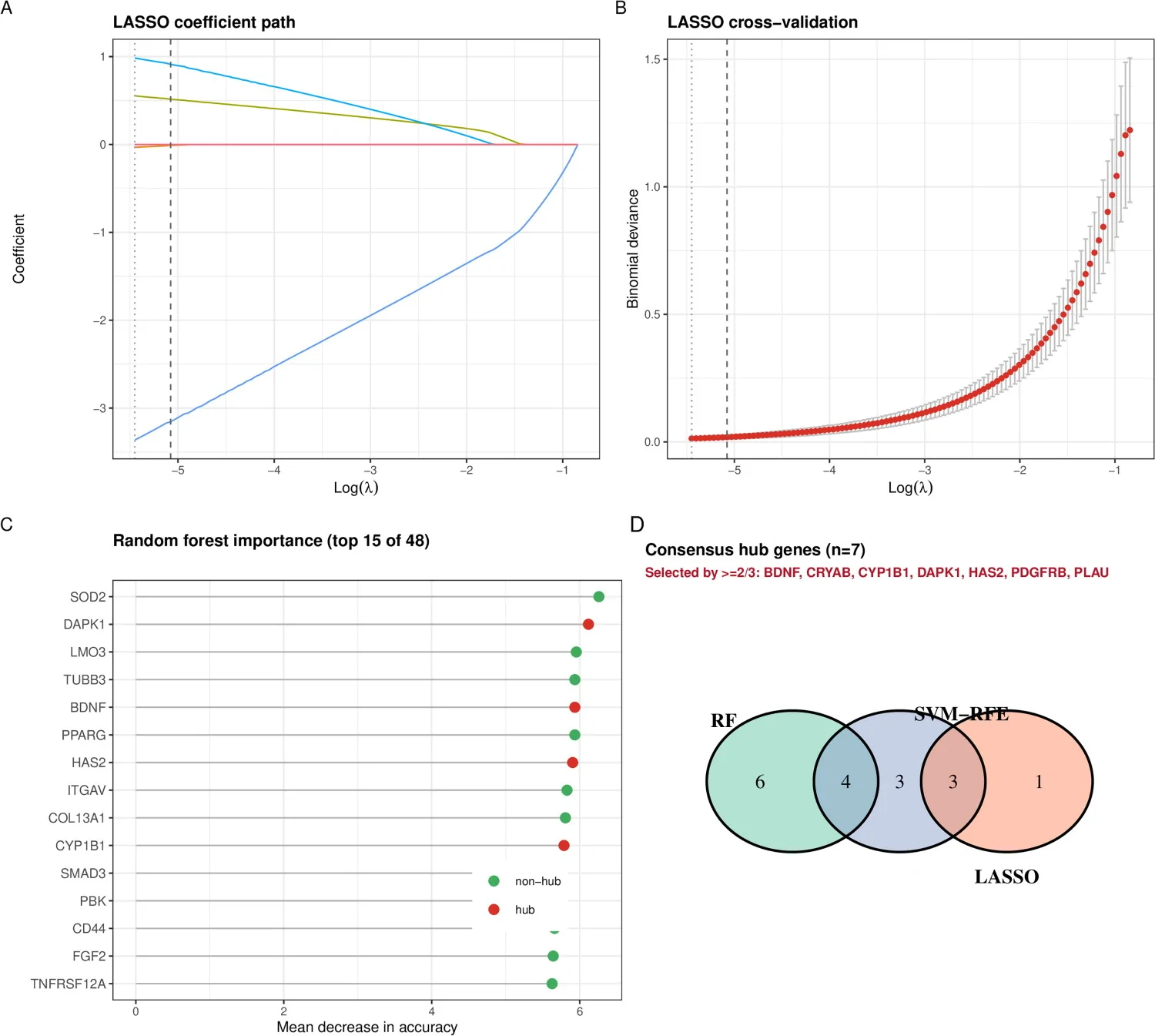
Symmetric machine-learning hub-gene selection. (A) LASSO coefficient path. (B) LASSO cross-validation; λ1se. (C) Random forest importance (MDA). (D) Consensus of LASSO, random forest, and SVM-RFE top-10 candidates; seven genes (BDNF, CRYAB, CYP1B1, DAPK1, HAS2, PDGFRB, PLAU) were selected by ≥ 2 of 3 algorithms. The full SVM-RFE ranking is in Supplementary Table S4.

Under the symmetric ≥2-of-3 consensus (top 10 per algorithm), LASSO (λ1se) retained PLAU, CRYAB, PDGFRB and BUB1 (Figure 4A,B); random-forest importance ranked by Mean Decrease Accuracy is shown in Figure 4C (top 15 of the 48 DEARGs displayed), and both the random-forest and SVM-RFE top-10 rankings are reported in full in Supplementary Table S4 (SVM-RFE ranks 1 and 2 were PLAU and CRYAB). Seven genes were selected by ≥ 2 of 3 algorithms: BDNF, CRYAB, CYP1B1, DAPK1, HAS2, PDGFRB, and PLAU (Figure 4D, Table 2). BUB1, a mitotic-checkpoint kinase [47] identified as a hub gene in the original analysis, was selected by only one algorithm under the corrected symmetric rule and was therefore not retained; S100A4 did not qualify.

**Table 2.** Hub anoikis-related genes identified by symmetric multi-algorithm consensus.

Gene	Direction (VC)	ML methods (≥2/3)	Note/known function
PLAU	Down	LASSO, SVM-RFE	Urokinase plasminogen activator; pericellular proteolysis; anoikis induction
CRYAB	Down	LASSO, SVM-RFE	αB-crystallin; chaperone; anti-apoptotic
PDGFRB	Down	LASSO, SVM-RFE	VSMC/pericyte lineage & survival receptor; lost in modulated VSMC
BDNF	—	RF, SVM-RFE	Neurotrophic factor; very low single-cell expression; role in VSMC uncertain
CYP1B1	—	RF, SVM-RFE	Cytochrome P450; oxidative-stress/vascular responses
DAPK1	—	RF, SVM-RFE	Death-associated protein kinase; pro-apoptotic
HAS2	—	RF, SVM-RFE	Hyaluronan synthase 2; ECM remodeling

Panel revised from four genes (BUB1, CRYAB, PDGFRB, PLAU) to seven genes under the corrected symmetric consensus; BUB1 was selected by only one algorithm and is no longer a hub gene. None of the panel genes belong to the 36 canonical GO anoikis genes.

### 3.7. Diagnostic model performance

**Figure 5.**
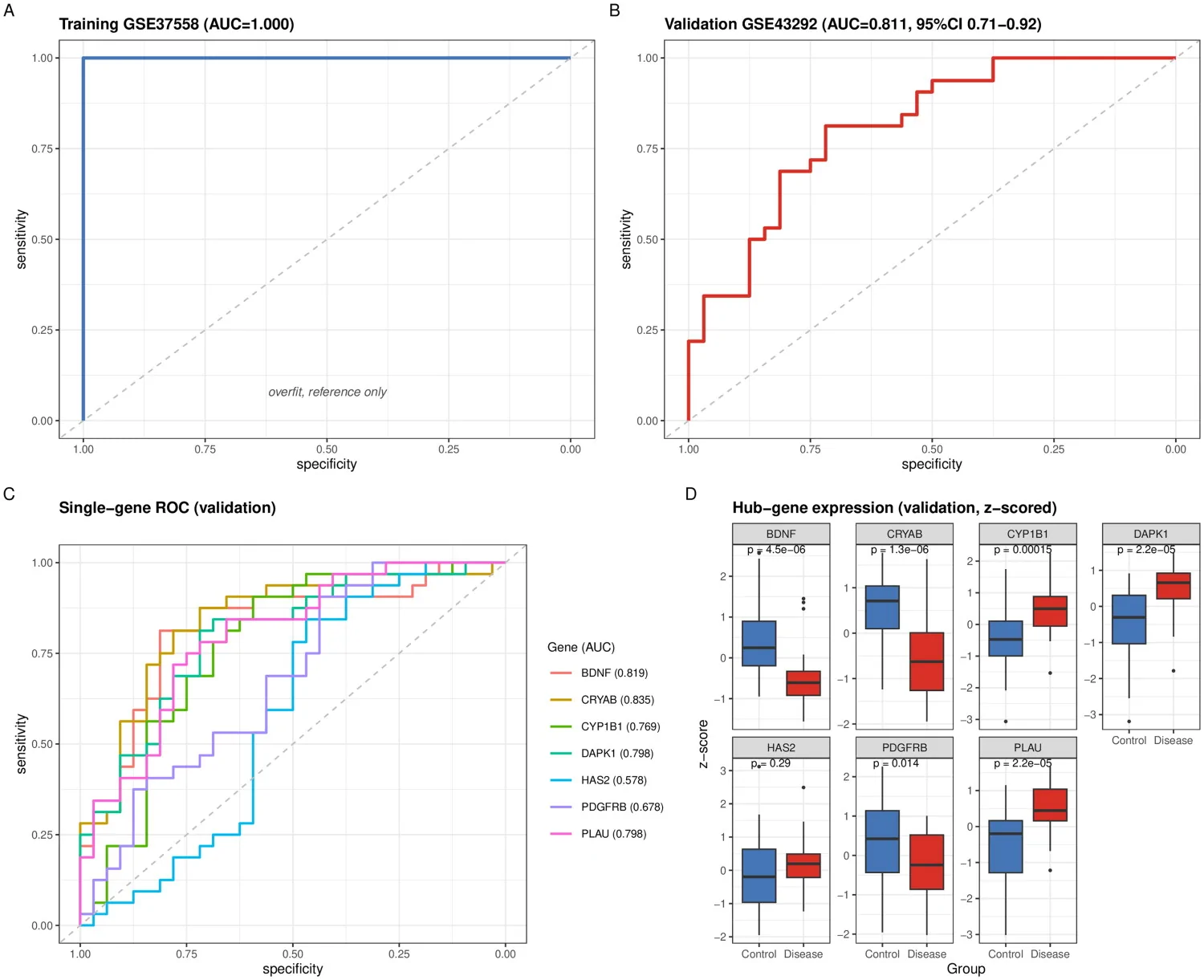
Diagnostic model performance. (A) Training ROC (GSE37558; apparent AUC = 1.000, not interpreted as performance). (B) External validation ROC (GSE43292; AUC = 0.811, DeLong 95% CI 0.706–0.915). (C) Single-gene validation ROC curves. (D) Hub-gene expression boxplots.

The seven-gene logistic model achieved an apparent training AUC of 1.000 in GSE37558. Because the panel genes were selected for differential expression in this same small cohort (*n* = 16), this apparent value—and resampling estimates derived from it (repeated 5-fold CV AUC ≈ 1.000)—are optimistically biased and are not interpreted as performance. Specifically, the bootstrap optimism-corrected AUC was likewise ≈1.00 and the Hosmer–Lemeshow test was non-informative under complete separation (*p* ≈ 1.0); these internal metrics are reported only for completeness and are not interpretable as performance. In the independent external cohort GSE43292 (*n* = 64), the model attained an AUC of 0.811 (DeLong 95% CI 0.706–0.915) (Figure 5A,B). Among single genes in the validation set, CRYAB (AUC 0.835), BDNF (0.819), DAPK1 (0.798) and PLAU (0.798) showed the strongest standalone discrimination (Figure 5C,D).

### 3.8. Hub gene–immune correlations

**Figure 6.**
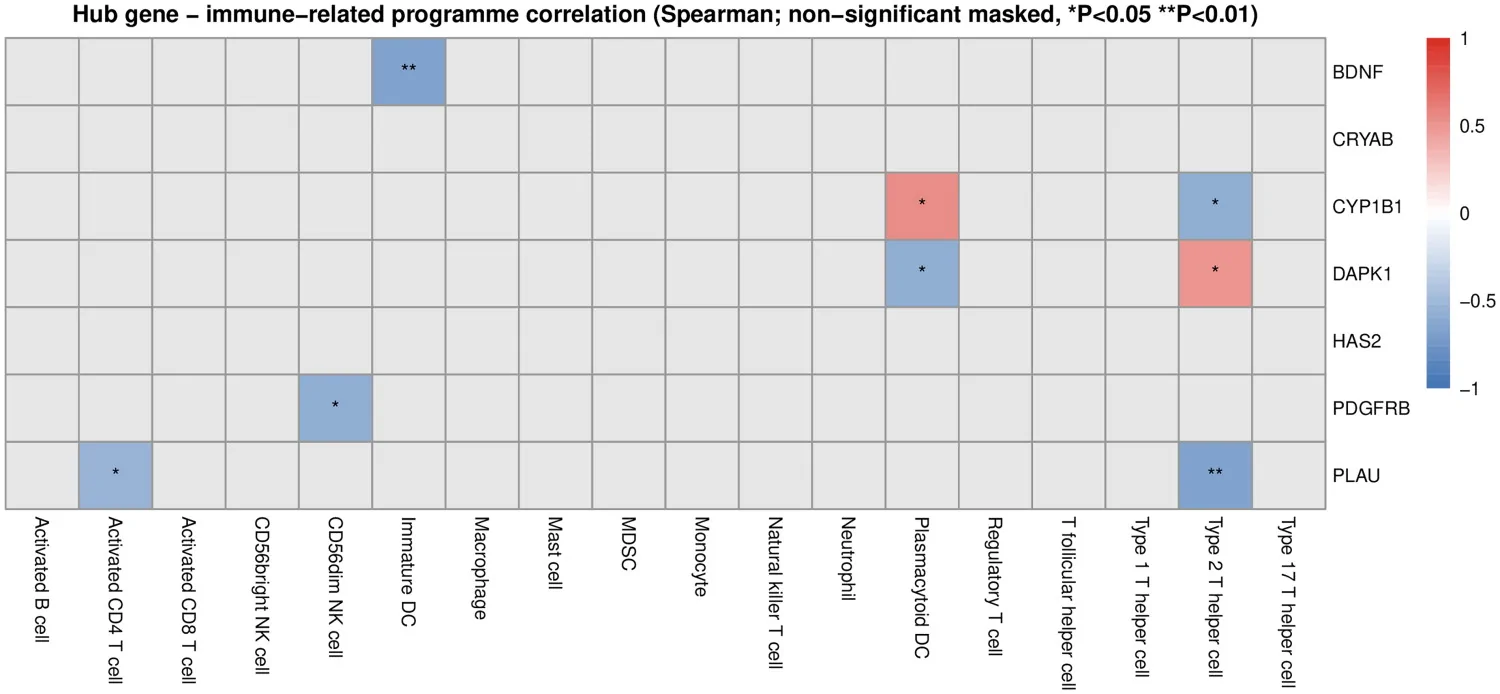
Hub gene–immune-related gene-programme correlation heatmap (Spearman; GSE37558). Non-significant correlations (*p* ≥ 0.05) are masked in grey; significant cells are annotated with asterisks (*p < 0.05, **p < 0.01).

Spearman analysis revealed several moderate hub-gene–immune-programme correlations in GSE37558 (e.g., PLAU and BDNF negatively correlated with Th2 and immature-DC programmes, respectively; Figure 6). As these derive from an in-vitro monoculture, they reflect co-regulation of immune-related gene modules rather than physical immune–VSMC interactions and require tissue-level validation.

### 3.9. PPI network topology

**Figure 7.**
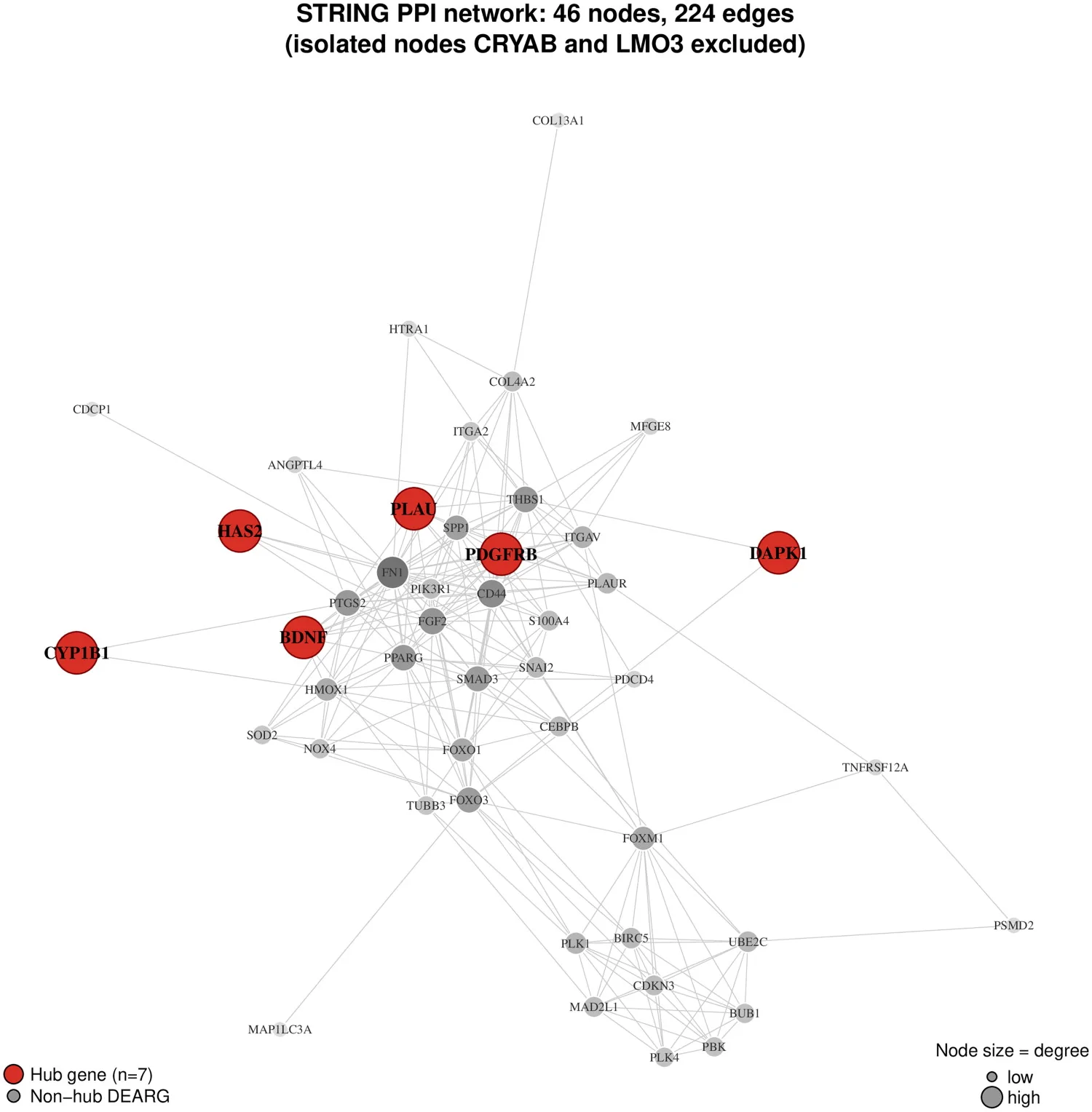
Protein–protein interaction (PPI) network of the 48 union DEARGs (STRING v12.0, confidence ≥ 0.400). Nodes are labeled with gene symbols; the seven consensus hub genes (BDNF, CRYAB, CYP1B1, DAPK1, HAS2, PDGFRB, PLAU) are highlighted in red and enlarged, while the remaining DEARGs are shaded grey by connectivity (node size scales with degree). The network comprises 46 nodes and 224 edges; the two isolated nodes CRYAB and LMO3, which had no medium-confidence interactions, were excluded from the displayed network. PDGFRB connects to FN1 and ITGAV within the network.

The STRING network from the 48 union DEARGs comprised 46 nodes and 224 edges; 2 genes (CRYAB and LMO3) had no medium-confidence interactions and were excluded from the network (Figure 7). PDGFRB connected to growth-factor-receptor and adhesion nodes including FN1 and ITGAV.

### 3.10. Single-cell contextualization of hub gene expression

**Figure 8.**
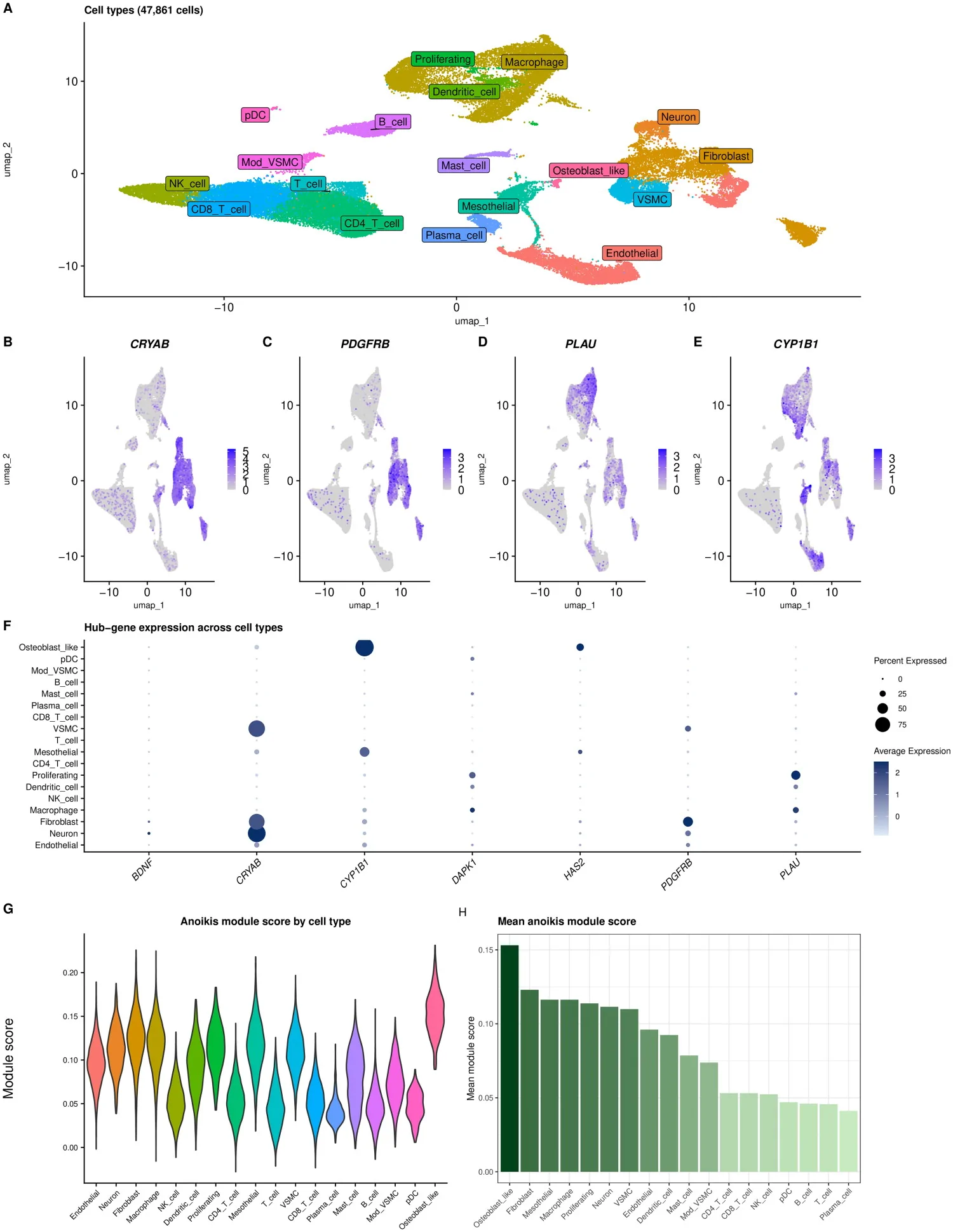
Single-cell contextualization in GSE159677. (A) UMAP of 47,861 cells annotated into 18 cell types (labels placed with repelled boxes). (B–E) FeaturePlots of representative hub genes (CRYAB, PDGFRB, PLAU, CYP1B1). (F) DotPlot of all seven hub genes across cell types; PDGFRB highest in VSMCs/fibroblasts and lost in modulated VSMCs and osteoblast-like cells. (G) Violin plots of the anoikis module score by cell type. (H) Mean anoikis module score per cell type; highest in osteoblast-like, fibroblast, mesothelial and macrophage populations, with comparatively low scores in modulated VSMCs.

Analysis of 47,861 cells from GSE159677 (18 annotated cell types; Figure 8A) showed distinct cell-type-specific hub-gene expression (Figure 8B–E): CRYAB was highest in contractile VSMCs, neurons and fibroblasts; PDGFRB was highest in VSMCs and fibroblasts and near-absent in modulated VSMCs and osteoblast-like cells, consistent with loss of contractile identity during phenotypic switching; PLAU was enriched in macrophages and proliferating cells; and CYP1B1 was most abundant in osteoblast-like and mesothelial cells. A dot plot of all seven hub genes across the 18 annotated cell types summarizes these patterns and further shows HAS2 largely restricted to osteoblast-like and mesothelial cells, DAPK1 to macrophages and proliferating cells, and BDNF expressed at very low levels in every population (Figure 8F).

Anoikis module scoring (ARGs detected in the dataset) was highest in osteoblast-like, fibroblast, mesothelial and macrophage populations, whereas modulated VSMCs showed comparatively low scores (Figure 8G–H). This pattern indicates that osteogenic and macrophage populations within the calcifying vascular wall display the most pronounced anoikis-related transcriptomic activity. Because GSE159677 derives from carotid atherosclerotic plaques without CKD background, these results contextualize hub-gene expression in vascular disease rather than validating CKD-VC-specific biology.

## 4. Discussion

In this study we performed a focused integrative transcriptomic and single-cell analysis of anoikis-related molecular signatures at the anoikis–vascular-calcification intersection. Although several regulated cell-death modalities have been implicated in VC, the role of anoikis—triggered specifically by loss of cell–ECM adhesion—had not been examined in this setting despite a strong biological rationale linking ECM remodeling, VSMC detachment, and phenotypic switching. Using two complementary models, machine learning, and single-cell contextualization, we nominate a seven-gene anoikis-related panel (BDNF, CRYAB, CYP1B1, DAPK1, HAS2, PDGFRB, PLAU) as candidate molecular signatures of phosphate-driven VSMC calcification. The seven-gene logistic model generalized to an independent vascular-disease cohort (external-validation AUC = 0.811, 95% CI 0.706–0.915). We emphasize that these findings are exploratory and hypothesis-generating rather than evidence of a clinically deployable diagnostic tool.

A central observation of our re-analysis concerns the cross-system nature of the signal. When the rat in-vivo dataset was analyzed rigorously (count-based voom on re-quantified reads, with formal one-to-one orthology mapping), it showed no statistical enrichment for anoikis-related genes (*p* = 0.84), and no anoikis gene was differentially expressed in both the rat and human datasets. The anoikis-related signal thus derives essentially from the human in-vitro high-phosphate VSMC model. Accordingly, the panel should be regarded as a candidate signal of shared phosphate-driven VSMC calcification programmes rather than a CKD-specific or cross-species-conserved molecular fingerprint, and the rat dataset is best viewed as a non-confirmatory in-vivo context.

It is also important to note that none of the panel genes belong to the 36 canonical GO-defined anoikis genes; they derive from the broader GeneCards keyword list. The panel therefore more accurately reflects general cell–matrix adhesion, apoptosis, and proliferation-survival programmes than strictly canonical anoikis mediators, and should be interpreted accordingly. Although the panel genes are not GO-core anoikis effectors, anoikis is operationally an adhesion-loss–triggered apoptotic programme; the convergence of our DEARGs and the top-ranked GSVA pathway on cell–ECM adhesion molecules (CAMs, adjusted *p* = 0.002) supports interrogation of this adhesion–survival axis as anoikis-relevant, while we explicitly refrain from claiming canonical anoikis-effector status.

Among the panel, PLAU has the most direct mechanistic link to anoikis: pericellular plasmin generated by urokinase degrades fibronectin and induces VSMC detachment and anoikis [26]. CRYAB (αB-crystallin) is a small heat-shock protein that inhibits caspase-3 maturation and provides chaperone-mediated cytoprotection [48–50]; its downregulation during calcification may sensitize VSMCs to stress-induced death. PDGFRB is a canonical VSMC/pericyte lineage and survival receptor [51]; consistent with phenotypic switching, single-cell data showed PDGFRB highest in VSMCs and fibroblasts and near-absent in modulated VSMCs and osteoblast-like cells. The newly identified candidates—BDNF, CYP1B1, DAPK1 and HAS2—are more weakly characterized in VSMC biology: DAPK1 is a pro-apoptotic kinase linked to anoikis; HAS2 (hyaluronan synthase 2) participates in ECM remodeling; CYP1B1 is implicated in oxidative-stress and vascular responses; and BDNF is a neurotrophic factor whose role in VSMCs is incompletely understood and which, notably, was expressed at very low levels in the single-cell data—warranting cautious interpretation. We refrain from over-interpreting these as specific anoikis effectors. More broadly, the proliferation–survival balance that these genes report on is itself relevant to vascular remodeling: VSMC populations expand oligoclonally during vascular disease [52], and chronic VSMC apoptosis is sufficient to accelerate atherosclerosis and calcification *in vivo* [53].

The relevance of the broader plasminogen axis to vascular calcification is supported by independent evidence: elevated soluble uPAR (encoded by PLAUR) causally promotes coronary artery calcification and atherosclerosis *via* monocyte-mediated inflammation [54], and suPAR is independently associated with cardiovascular calcification in dialysis patients [55,56]. While PLAUR itself was not part of the final panel, the prominence of PLAU is consistent with transcriptional perturbation of the urokinase system during vascular calcification. These observations sit within a broader inflammatory axis in CKD-associated VC, in which pro-inflammatory signaling—interleukin-6 in particular—promotes VSMC osteogenic transition and mineralization [57,58].

The external validation cohort (GSE43292) represents carotid atherosclerotic intimal calcification, which is mechanistically distinct from CKD-associated medial calcification: intimal calcification is lipid- and inflammation-driven, whereas CKD medial calcification is driven by mineral-metabolism disturbance and VSMC osteogenic transdifferentiation [6,8, 59,60]. The model was therefore tested for generalization to a distinct vascular-calcification context rather than validated for CKD-VC; a dedicated CKD-VC cohort remains lacking. Similarly, the single-cell dataset derives from atherosclerotic plaques without CKD background and provides contextualization rather than CKD-VC validation.

Several limitations should be acknowledged. First, the training datasets are small (*n* = 14 and 16) and derive from different species, platforms, and biological systems (in-vivo rat aorta vs in-vitro human VSMC); the anoikis signal was statistically supported only in the human in-vitro dataset. Second, with only 16 training samples and seven correlated predictors, the model perfectly separates the training data; internal resampling estimates are consequently optimistically biased, and only the external-validation AUC should be interpreted as evidence of generalizability. Third, the ARG set is broad and keyword-derived, and the panel genes are not canonical anoikis genes. Fourth, GSE43292 and GSE159677 represent atherosclerotic rather than CKD-VC tissue. Fifth, the study is entirely computational; functional experiments—e.g., PLAU or CRYAB modulation in high-phosphate/uremic-toxin VSMC models with measurement of detachment-induced apoptosis, caspase activity, and mineralization—are required before any causal or mechanistic conclusion can be drawn.

## 5. Conclusions

This study provides a focused, hypothesis-generating characterization of anoikis-related molecular signatures at the intersection of anoikis and vascular calcification. Using integrative transcriptomic analysis, a pre-specified symmetric machine-learning consensus, and single-cell contextualization, we nominate seven candidate genes—BDNF, CRYAB, CYP1B1, DAPK1, HAS2, PDGFRB, and PLAU—linked to chaperone-mediated cytoprotection, VSMC lineage maintenance, and pericellular proteolytic remodeling. The signature was supported by the human in-vitro VSMC model and generalized to an independent atherosclerotic cohort (external-validation AUC = 0.811), but was not corroborated by the rat in-vivo dataset and is not anchored in canonical anoikis genes. These candidate molecular signatures require validation in CKD-specific vascular tissue and functional experiments before any diagnostic or therapeutic application can be considered.

## Supplementary Material

Supplementary Material.docx

## Data Availability

All underlying data and analytic code supporting this study are openly available in a Zenodo repository (https://doi.org/10.5281/zenodo.20507232). The deposit contains the gene-level count matrix for GSE146638 re-quantified from SRA (which GEO does not provide), all intermediate result tables, the complete analysis and figure-generation code (re-quantification pipeline SRA→salmon→counts, ortholog mapping, differential expression, machine-learning, and validation), and high-resolution figures. The primary source datasets are publicly available from GEO (GSE146638, GSE37558, GSE43292, GSE159677) and SRA (study SRP252073).
