# Supplementary material for: Integrative transcriptomic and single-cell analysis identifies anoikis-related molecular signatures in vascular calcification
Source: Ren Fail. 2026 Aug 24;48(1):2704989. doi: 10.1080/0886022X.2026.2704989 (PMC13508498; doi:10.1080/0886022X.2026.2704989)
Supplement: Supplementary file 1 [file IRNF_A_2704989_SM0186.docx]

**Supplementary Material**

**Supplementary Methods / Results**

**§S2. Re-quantification of GSE146638 from raw reads**

GEO provides only RPKM values for GSE146638 (rat, Rattus norvegicus, rn5). Raw single-end reads (SRA study SRP252073; 30 runs for 14 samples) were adapter/quality-trimmed (fastp), quantified against the Ensembl release-110 rat transcriptome (salmon, mapping rate 45–53%), and summarized to gene-level counts (tximport; 29,910 genes). Differential expression used limma-voom (filterByExpr retained 12,819 genes); DESeq2 recovered 88% of voom DEGs.

**§S3. ARG-set sensitivity analysis**

The 529-gene ARG set comprises 36 GO:BP core anoikis genes plus GeneCards keyword genes (relevance > 1.0). Restricting to the 36 GO-core genes reduced the union DEARG set to 1 gene; a stricter GeneCards threshold (>7.0) gave 1 gene. None of the seven panel genes (BDNF, CRYAB, CYP1B1, DAPK1, HAS2, PDGFRB, PLAU) belong to the 36 GO-core anoikis genes; all derive from the broader GeneCards list. The panel therefore reflects general cell–matrix adhesion, apoptosis, and proliferation-survival programmes rather than canonical anoikis mediators. Full annotated ARG list: Supplementary Table S2.

**§S4. Enrichment of rat vs human datasets for ARGs**

Hypergeometric testing (background ≈ 20,000 genes): rat-derived DEGs were not enriched for ARGs (2 observed vs 3.2 expected; P = 0.84), whereas human DEGs were strongly enriched (46 vs 18.3; P = 9.5×10⁻⁹). This held across alternative rat groupings (Control vs Uremic; Control vs VitD3) and thresholds. The anoikis-related signal therefore derives from the human in-vitro VSMC model.

**§S5. Immune-related gene-programme analysis (ssGSEA)**

ssGSEA of 18 immune-cell gene programmes (Charoentong et al.) in GSE37558 showed only nominal (uncorrected) trends (e.g., Th2, pDC); no programme survived FDR correction (best FDR ≈ 0.79). Because GSE37558 is an in-vitro VSMC monoculture devoid of immune cells, these scores reflect immune-related gene-expression reprogramming within VSMCs, not immune-cell infiltration.

**Supplementary Tables (provided in Supplementary-Tables-S1-S5.xlsx)**

Table S1. CKD-VC-relevant datasets considered and inclusion/exclusion reasons.

Table S2. Full 529-gene anoikis-related gene (ARG) set with source annotation (GO-core vs GeneCards) and relevance score.

Table S3. DEARG lists: union (48), rat-derived (2), human (46); cross-species common = 0.

Table S4. Complete SVM-RFE ranking of the 48 candidate genes (ranks 1–2 = PLAU, CRYAB).

Table S5. The 15 a-priori KEGG pathways analysed by GSVA, with logFC and FDR.

**Supplementary Figure Legends**

Figure S0. Analysis pipeline schematic (re-quantification → voom DE → orthology → DEARG → ML consensus → validation → single-cell).

Figure S3. ARG-set sensitivity: union DEARG size under the full 529 set vs 36 GO-core vs GeneCards>7.0.

Figure S5. ssGSEA immune-related gene-programme scores in GSE37558 (no programme significant after FDR).
